# An Overview on Wireless Sensor Networks Technology and Evolution

**DOI:** 10.3390/s90906869

**Published:** 2009-08-31

**Authors:** Chiara Buratti, Andrea Conti, Davide Dardari, Roberto Verdone

**Affiliations:** 1 WiLAB, DEIS at University of Bologna, Bologna, Italy; E-Mails: ddardari@ieee.org (D.D.); roberto.verdone@unibo.it (R.V.); 2 WiLAB, ENDIF at University of Ferrara, Ferrara, Italy; E-Mail: a.conti@ieee.org (A.C.)

**Keywords:** wireless sensor networks, enabling technologies, applications, IEEE 802.15.4

## Abstract

Wireless sensor networks (WSNs) enable new applications and require non-conventional paradigms for protocol design due to several constraints. Owing to the requirement for low device complexity together with low energy consumption (i.e., long network lifetime), a proper balance between communication and signal/data processing capabilities must be found. This motivates a huge effort in research activities, standardization process, and industrial investments on this field since the last decade. This survey paper aims at reporting an overview of WSNs technologies, main applications and standards, features in WSNs design, and evolutions. In particular, some peculiar applications, such as those based on environmental monitoring, are discussed and design strategies highlighted; a case study based on a real implementation is also reported. Trends and possible evolutions are traced. Emphasis is given to the IEEE 802.15.4 technology, which enables many applications of WSNs. Some example of performance characteristics of 802.15.4-based networks are shown and discussed as a function of the size of the WSN and the data type to be exchanged among nodes.

## 1. Introduction

Since the start of the third Millennium, wireless sensor networks (WSNs) generated an increasing interest from industrial and research perspectives [1–7]. A WSN can be generally described as a network of nodes that cooperatively sense and may control the environment enabling interaction between persons or computers and the surrounding environment [8]. On one hand, WSNs enable new applications and thus new possible markets, on the other hand, the design is affected by several constraints that call for new paradigms. In fact, the activity of sensing, processing, and communication under limited amount of energy, ignites a cross-layer design approach typically requiring the joint consideration of distributed signal/data processing, medium access control, and communication protocols [9].

This paper provides a survey of WSNs technologies, main applications and standards, features in WSNs design with case study, and evolutions. In particular example of performance based on experimental results will be reported. With respect to the literature [1, 2, 10] this paper deals not only with applications and features of WSNs, or only on design of WSNs, but puts together all these aspects, focusing also the attention on technologies and standards.

WSNs have several common aspects with wireless ad hoc network [11] and in many cases they are simply considered as a special case of them. This could be lead to erroneous conclusions, especially when protocols and algorithms designed for ad hoc networks are used in WSN. For this reason in Section 2 an appropriate definition of WSN and discussion is provided.

In Section 3, the main application areas for WSNs are categorized according to the type of information measured or carried by the network. Applications, on top of the stack, set requirements that drive the selection of protocols and transmission techniques; at the other end, the wireless channel poses constraints to the communication capabilities and performance. Based on the requirements set by applications and the constraints posed by the wireless channel, the communication protocols and techniques are selected.

The main features in WSNs design are described in Section 4. Specifically, the design of energy efficient communication protocols is a very peculiar issue of WSNs, without significant precedent in wireless network history. Generally, when a node is in transmit mode, the transceiver drains much more current from the battery than the microprocessor in active state or the sensors and the memory chip. The ratio between the energy needed for transmitting and for processing a bit of information is usually assumed to be much larger than one (more than one hundred or one thousand in most commercial platforms). For this reason, the communication protocols need to be designed according to paradigms of energy efficiency, while this constraint is less restrictive for processing tasks. Then, the design of energy efficient communication protocols is a very peculiar issue of WSNs, without significant precedent in wireless network history. Most of the literature on WSNs deals with the design of energy efficient protocols, neglecting the role of the energy consumed when processing data inside the node, and conclude that the transceiver is the part responsible for the consumption of most energy. On the other hand, data processing in WSNs may require consuming tasks to be performed at the microprocessor, much longer than the actual length of time a transceiver spends in transmit mode. This can cause a significant energy consumption by the microprocessor, even comparable to the energy consumed during transmission, or reception, by the transceiver. Thus, the general rule that the design of communication protocol design is much more important than that of the processing task scheduling is not always true.

Some examples of network design are given in Sections 4.1 and 4.2, where a case study developed for environmental monitoring is reported.

The process of standardization in the field of WSNs is very active in the last years and an important outcome is represented by IEEE 802.15.4 which is a short-range communication system intended to provide applications with relaxed throughput and latency requirements in Wireless Personal Area Networks (WPAN) [12]. The main features of the 802.15.4 standard are resumed in Section 5, where examples of performance indexes are illustrated in terms of area throughput and energy efficiency. Others technologies such as UltraWideBand (UWB), Bluetooth and other custom-defined technologies are reported in Section 6. We finally conclude the paper by giving our vision on future research directions in Section 7.

## 2. Wireless Sensor Networks

A WSN can be defined as a network of devices, denoted as *nodes*, which can sense the environment and communicate the information gathered from the monitored field (e.g., an area or volume) through wireless links [1–9]. The data is forwarded, possibly via multiple hops, to a *sink* (sometimes denoted as *controller* or *monitor*) that can use it locally or is connected to other networks (e.g., the Internet) through a *gateway*. The nodes can be stationary or moving. They can be aware of their location or not. They can be homogeneous or not.

**Figure 1.**
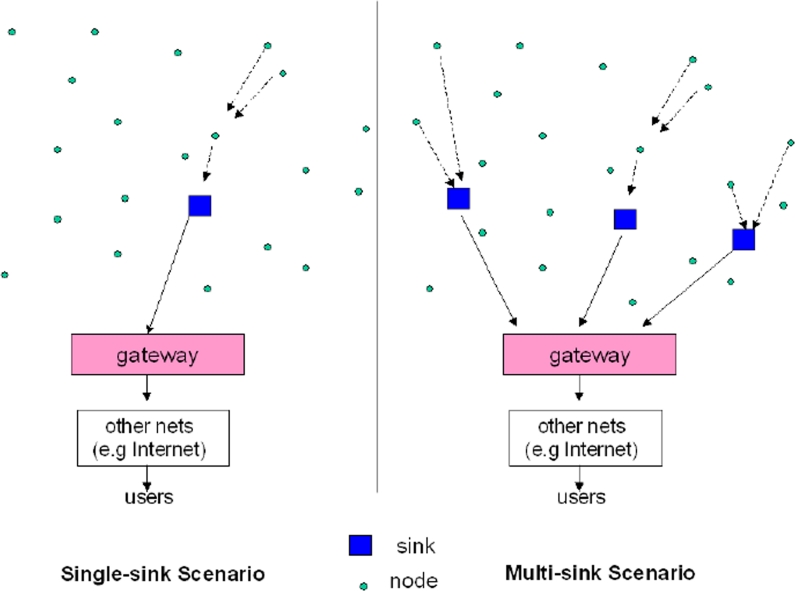
Left part: single-sink WSN. Right part: multi-sink scenario.

This is a traditional single-sink WSN (see Figure 1, left part). Almost all scientific papers in the literature deal with such a definition. This single-sink scenario suffers from the lack of scalability: by increasing the number of nodes, the amount of data gathered by the sink increases and once its capacity is reached, the network size cannot be augmented. Moreover, for reasons related to MAC and routing aspects, network performance cannot be considered independent from the network size.

A more general scenario includes multiple sinks in the network (see Figure 1, right part) [13]. Given a level of node density, a larger number of sinks will decrease the probability of isolated clusters of nodes that cannot deliver their data owing to unfortunate signal propagation conditions. In principle, a multiple-sink WSN can be scalable (i.e., the same performance can be achieved even by increasing the number of nodes), while this is clearly not true for a single-sink network. However, a multi-sink WSN does not represent a trivial extension of a single-sink case for the network engineer. In many cases nodes send the data collected to one of the sinks, selected among many, which forward the data to the gateway, toward the final user (see Figure 1, right part). From the protocol viewpoint, this means that a selection can be done, based on a suitable criterium that could be, for example, minimum delay, maximum throughput, minimum number of hops, etc. Therefore, the presence of multiple sinks ensures better network performance with respect to the single-sink case (assuming the same number of nodes is deployed over the same area), but the communication protocols must be more complex and should be designed according to suitable criteria.

## 3. Applications of WSNs

The variety of possible applications of WSNs to the real world is practically unlimited, from environmental monitoring [14], health care [15], positioning and tracking [16], to logistic, localization, and so on. A possible classification for applications is provided in this section.

It is important to underline that the application strongly affects the choice of the wireless technology to be used. Once application requirements are set, in fact, the designer has to select the technology which allows to satisfy these requirements. To this aim the knowledge of the features, advantages and disadvantages of the different technologies is fundamental.

Owing to the importance of the relationship between application requirements and technologies, we report in this Section some example requirements and we devoted Sections 5 and 6 to an overview of the main features of the most promising technologies provided for WSNs.

### 3.1. Applications Classification

One of the possible classifications distinguishes applications according to the type of data that must be gathered in the network. Almost any application, in fact, could be classified into two categories: event detection (ED) and spatial process estimation (SPE).

In the first case sensors are deployed to detect an event, for example a fire in a forest, a quake, etc. [17–19]. Signal processing within devices is very simple, owing to the fact that each device has to compare the measured quantity with a given threshold and to send the binary information to the sink(s). The density of nodes must ensure that the event is detected and forwarded to the sink(s) with a suitable probability of success while maintaining a low probability of false alarm. The detection of the phenomenon of interest (POI) could be performed in a decentralized (or distributed) way, meaning that sensors, together with the sink, cooperatively undertake the task of identifying the POI. However, unlike in classical decentralized detection problems, greater challenges exist in a WSN setting. There are stringent power constraints for each node, communication channels between nodes and the fusion center are severely bandwidth-constrained and are no longer lossless (e.g, fading, noise and, possibly, external sources of interference are present), and the observation at each sensor node is spatially varying. In the context of decentralized detection, cooperation allows exchange of information among sensor nodes to continuously update their local decisions until consensus is reached across the nodes.

In SPE the WSN aims at estimating a given physical phenomenon (e.g., the atmospheric pressure in a wide area, or the ground temperature variations in a small volcanic site), which can be modelled as a bi-dimensional random process (generally non-stationary). In this case the main issue is to obtain the estimation of the entire behavior of the spatial process based on the samples taken by sensors that are typically placed in random positions [20–23]. The measurements will then subject to proper processing which might be performed either in a distributed manner by the nodes, or centrally at the supervisor. The estimation error is strictly related to nodes density as well as on the spatial variability of the process. Higher nodes density lead to a more accurate scalar field reconstruction at the expense of a larger network throughput and cost.

In the recent literature, different works addressed the estimation of a scalar field using random WSNs. As an example, [20] presents a distributed algorithm able to estimate the gradient of a generic smooth physical process (energy constraints and nodes failure are not considered there); in [21] the relationship between the random topology of a sensor network and the quality of the reconstructed field is investigated and some guidelines on how sensors should be deployed over a spatial area for efficient data acquisition and reconstruction are derived. Distributed source coding techniques can be successfully exploited to reduce the amount of data to be transmitted and hence to improve the network energy efficiency [24].

**Figure 2.**
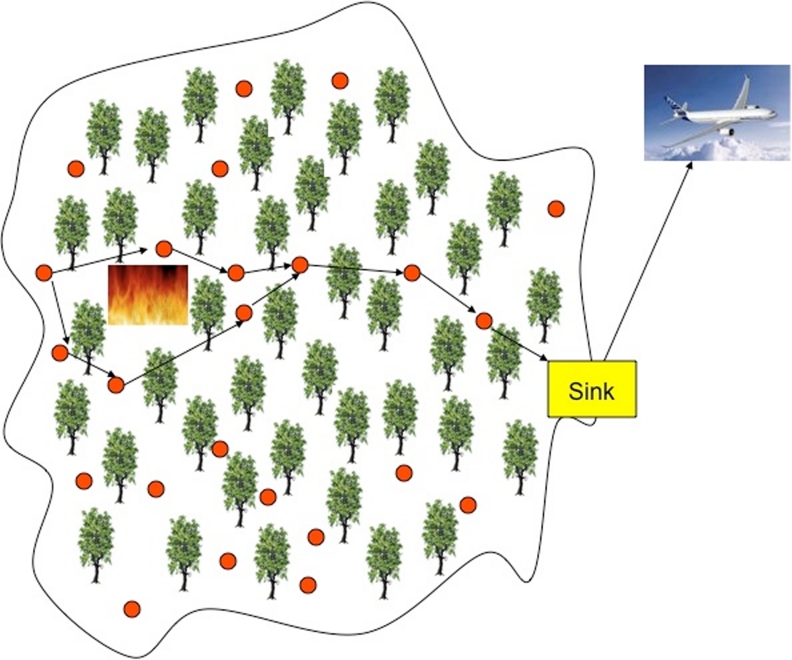
Event detection application.

There exist also applications that belong to both categories. As an example, environmental monitoring applications could be ED- or SPE-based. To the first category belong, for example, the location of a fire in a forest, or the detection of a quake, etc. (see Figure 2). Alternatively, the estimation of the temperature of a given area belongs to the second category. In general, these applications aim at monitoring indoor or outdoor environments, where the supervised area may be few hundreds of square meters or thousands of square kilometers, and the duration of the supervision may last for years. Natural disasters such as floods, forest fires, earthquakes may be perceived earlier by installing networked embedded systems closer to places where these phenomena may occur. Such systems cannot rely on a fixed infrastructure and have to be very robust, because of the inevitable impairments encountered in open environments. The system should respond to environment changes as quick as possible. The environment to be observed will mostly be inaccessible by the human all the time. Hence, robustness plays an important role. Also security and surveillance applications have some demanding and challenging requirements such as real-time monitoring and high security.

An other application that could belong to both the above defined categories is devoted to the realisation of energy efficient buildings. In this application, in fact, sensor nodes could aim at estimating a process (SPE), but also events (ED). In this case the WSN is distributed in buildings (residential or not) to manage efficiently the energy consumption of all the electric appliances. Consequently, nodes have to continuously monitor the energy consumed by all appliances connected to the electrical grid. Therefore, sensors have to estimate a process, that is the energy consumption which varies with time, but in some cases, they could be used to detect some events. As an example, sensors could detect the arrival of a person in a room to switch on some electrical appliances.

The European Joint Undertaking ARTEMISIA has funded the project eDIANA (Embedded Systems for Energy Efficient Buildings), focused on the above described application scenario. The project, in fact, aims at achieving energy efficient buildings through innovative solutions based on networked embedded systems. The eDIANA approach is to achieve greater efficiency in the use of resources, prioritizing energy as scarce resource, more flexibility in the provision of resources and better situation awareness for the citizen and for service and infrastructure owners. This will be achieved through the deployment and inter-operation of embedded systems throughout the eDIANA environment of buildings and intra-building units.

### 3.2. Examples of Application Requirements

Due to the wide variety of possible applications of WSNs, system requirements could change significantly. For instance, in case of environmental monitoring applications, the following requirements are typically dominant: *energy efficiency*, nodes are battery powered or have a limited power supply; *low data rate*, typically the amount of data to be sensed is limited; *one-way communication*, nodes act only as sensors and hence the data flow is from nodes to sink(s); *wireless backbone*, usually in environmental monitoring no wired connections are available to connect sink(s) to the fixed network.

Significantly different are the requirements of a typical industrial application where wireless nodes are used for cable replacement: *reliability*, communication must be robust to failure and interference; *security*, communication must be robust to intentional attacks; *inter-operability*, standards are required; *high data rate*, the process to be monitored usually carries a large amount of data; *two-way communication*, in industrial applications nodes typically act also as actuators and hence the communication between sink(s) and nodes must be guaranteed; *wired backbone*, sinks can be connected directly to the fixed network using wired connections.

Even if requirements are strongly application dependent, one of the most important issues in the design of WSNs, especially in such scenarios where power supply availability is limited, is *energy efficiency*. High energy efficiency means long network lifetime and limited network deployment and maintenance costs.

Energy efficiency can be achieved at different levels starting from the technology level (e.g., by adopting low consumption hardware components), physical layer, MAC, routing protocols up to the application level.

For example, at physical and MAC layers, nodes could operate with low duty cycle by spending most of their time in sleeping mode to save energy. This poses new problems such as that nodes may not wake up at the same time, due to the drifts of their local clocks, thus making the communication impossible. Suitable network synchronization schemes are mandatory in this case [8, 25].

## 4. Main Features in Wireless Sensor Networks Design

The main features of WSNs, as could be deduced by the general description given in the previous sections, are: scalability with respect to the number of nodes in the network, self-organization, self-healing, energy efficiency, a sufficient degree of connectivity among nodes, low-complexity, low cost and size of nodes. Those protocol architectures and technical solutions providing such features can be considered as a potential framework for the creation of these networks, but, unfortunately, the definition of such a protocol architecture and technical solution is not simple, and the research still needs to work on it [5].

The massive research on WSNs started after the year 2000. However, it took advantage of the outcome of the research on wireless networks performed since the second half of the previous century. In particular, the study of ad hoc networks attracted a lot of attention for several decades, and some researchers tried to report their skills acquired in the field of ad hoc networks, to the study of WSNs.

According to some general definitions, wireless ad hoc networks are formed dynamically by an autonomous system of nodes connected via wireless links without using an existing network infrastructure or centralized administration. Nodes are connected through “ad hoc” topologies, set up and cleared according to user needs and temporary conditions [11]. Apparently, this definition can include WSNs. However, this is not true. This is the list of main features for wireless ad hoc networks: unplanned and highly dynamical; nodes are “smart” terminals (laptops, etc.); typical applications include realtime or non-realtime data, multimedia, voice; every node can be either source or destination of information; every node can be a router toward other nodes; energy is not the most relevant matter; capacity is the most relevant matter [11].

Apart from the very first item, which is common to WSNs, in all other cases there is a clear distinction between WSNs and wireless ad hoc networks. In WSNs, nodes are simple and low-complexity devices; the typical applications require few bytes sent periodically or upon request or according to some external event; every node can be either source or destination of information, not both; some nodes do not play the role of routers; energy efficiency is a very relevant matter, while capacity is not for most applications. Therefore, WSNs are not a special case of wireless ad hoc networks. Thus, a lot of care must be used when considering protocols and algorithms which are good for ad hoc networks, and using them in the context of WSNs.

### 4.1. Example of Wireless Sensor Network Design

Owing to the plethora of features, WSNs design involves a wide range of aspects and considerations and often imposes that several issues, like connectivity, access to the channel, signal processing techniques, etc., must be accounted for together.

As an example, in [24] a self-organizing single-sink WSN, enabling environmental monitoring through the estimate of a scalar field over a bi-dimensional scenario, is considered. Nodes are assumed to be distributed according to a Poisson point process (PPP) over the area and are organized in a cluster-based topology. Connectivity issues, randomness of the channel, MAC issues and the role of distributed digital signal processing (DDSP) techniques are jointly accounted for, in a mathematical framework developed in the paper. Owing to the requirement of low device complexity together with low energy consumption (i.e., long network lifetime), a proper balance between communication and signal processing capabilities must be found. The adoption of DDSP techniques aims at reducing the amount of transmitted data over the wireless medium; on the other hand, the complexity of the signal processing performed at a single node has to be kept under control [26–28]. In [24] the possibility that nodes perform DDSP is studied through a distributed compression technique based on signal re-sampling. The DDSP impact on network energy efficiency is compared through a novel mathematical approach to the case where the processing is performed entirely by the sink.

The model developed allows the analysis of the network under two different perspectives: the estimation of the process and the energy consumption. The trade-off between energy conservation and estimation error is discussed and a design criterion proposed.

As an example result, the required node density is found as a trade-off between estimation quality and network lifetime for different system parameters and scalar field characteristics. It is shown that both the DDSP technique and the MAC protocol choice has a relevant impact on the performance of a WSN.

The main goal of [24] is neither to design specific communication protocols, nor DDSP techniques; rather, the joint consideration of all aspects mentioned, under realistic but simple working conditions, aims at stressing their interdependencies in a formalized framework.

Therefore, being the goal of [24], the proposal of a new approach for designing WSNs suffers the following limits: (i) a single-sink scenario and not the more general multi-sink scenario, is accounted for; (ii) the MAC protocol is very simple (slotted ALOHA), and no reference to any specific standard air interface is provided.

In [29–31] a multi-sink WSN, collecting data from the environment through the sampling of some physical entities and sending them to some external user, through multiple sinks, is considered. Through a simple polling model, sinks periodically issue queries, causing all sensors perform sensing and communicating their measurement results back to the sinks they are associated with. IEEE 802.15.4-compliant devices are accounted for and nodes access the channel through the CSMA-based MAC protocol described in Section 5. This paper introduces the concept of *area throughput*, that is, the amount of data per second successfully transmitted to the sinks from a given area. This performance metric is strictly related to both connectivity and MAC issues: it depends, in fact, on the probability that a given sensor node is not isolated and that it succeeds in transmitting its packet (i.e., the packet does not collide).

### 4.2. Case Study: Environmental Monitoring

**Figure 3.**
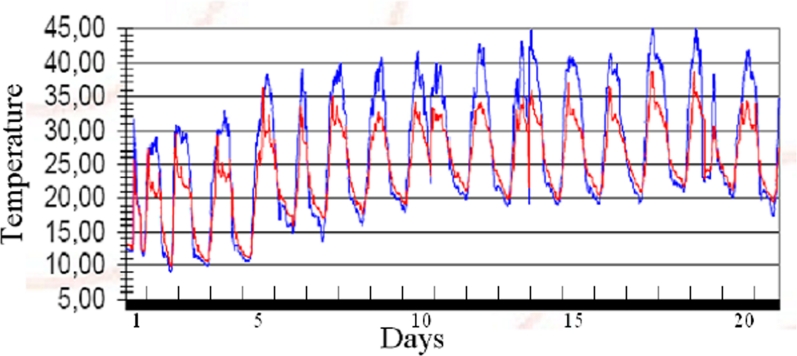
Screenshot of temperature behavior measured during June 2006 by 2 different nodes (red and blue curves, respectively).

In this section a low-cost hardware and software WSN test-bed developed for agricultural monitoring is described. The platform has been designed to provide the farmer with a periodic and punctual monitoring of physical parameters (e.g., temperature, air pressure, humidity) for a realtime control of different micro-climates in a cultivation. Thanks to this information it is possible to increase the quality and amount of production, cut costs, and reduce the pollution caused by weed-killers. The test-bed developed in cooperation with the start-up company SeNet s.r.l. has been working since 2006 in a peach field located in Italy. Each node is composed of an IEEE 802.15.4 radio transceiver based on Chipcon CC2430 operating in the 2.4 GHz band, photovoltaic panels, rechargeable batteries, temperature and humidity sensors. Each node emits a maximum power equal to 0 dBm and it is capable of communicating with neighbor nodes up to a maximum distance of 100 meters in line-of-sight conditions. The communication protocol has been designed to allow an extremely low duty cycle where nodes wake up for 10 seconds (activity mode) at intervals of 15 minutes. During the activity mode, each node collects measured data from its sensors and transmits the information to the sink node. In case the sink node is out of the radio link range, data are forwarded by intermediate nodes, which act as relays, in a multi-hop fashion according to a mesh network topology. To this purpose, a robust ad hoc network synchronization protocol has been designed to compensate relative clock drifts among nodes thus avoiding that nodes wake up in not overlapped time intervals. In sleeping mode, node consumption is only 0.5 mA, whereas during the activity mode the overall consumption is 30 mA. The resulting network lifetime is in the order of several weeks (in the absence of photovoltaic panels) and it is limited only by the rechargeable battery lifetime when photovoltaic panels are present. All sensed data collected by the sink node (coordinator) are then forwarded to the Internet every 2 hours through a GPRS link. The periodic report as well as each node status can be examined through a remote standard Internet connection. In Figure 3 an example of data report related to the temperature behavior measured in June 2006 is shown.

**Figure 4.**
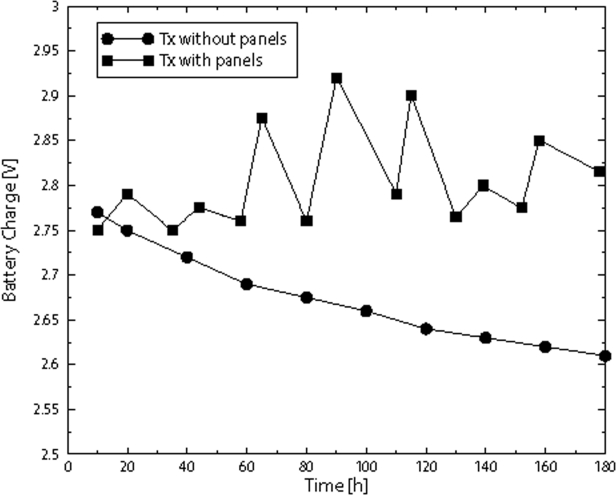
The behavior of the battery charge in Volt by passing time, expressed in hours, when photovoltaic panels are used and not.

Being energy efficiency one of the most important requirement for this application, we show the behavior of the battery status. In particular, in Figure 4 we show the battery charge in Volt as a function of time, expressed in hours, when photovoltaic panels are used and not. Two square panels with side of 10 cm are used.

## 5. IEEE 802.15.4 Technology

IEEE 802.15.4 wireless technology is a short-range communication system intended to provide applications with relaxed throughput and latency requirements in WPAN. The key features of 802.15.4 wireless technology are low complexity, low cost, low power consumption, low data rate transmissions, to be supported by cheap either fixed or moving devices. The main field of application of this technology is the implementation of WSNs.

The IEEE 802.15.4 Working Group (see also the website: http://www.ieee802.org/15/pub/TG4.html) focuses on the standardization of the bottom two layers of ISO/OSI protocol stack. There are two options for the upper layers definition: Zigbee protocols, specified by the industrial consortia ZigBee Alliance (see also the website: http://www.zigbee.org/en/index.asp) and 6LowPAN.

In the following, some technical details related to the physical and MAC layers as defined in the standard are reported. Finally some characteristics related to higher layers will be presented, considering Zigbee and 6LowPan, with particular attention to the former.

### 5.1. IEEE 802.15.4 Physical Layer

**Figure 5.**
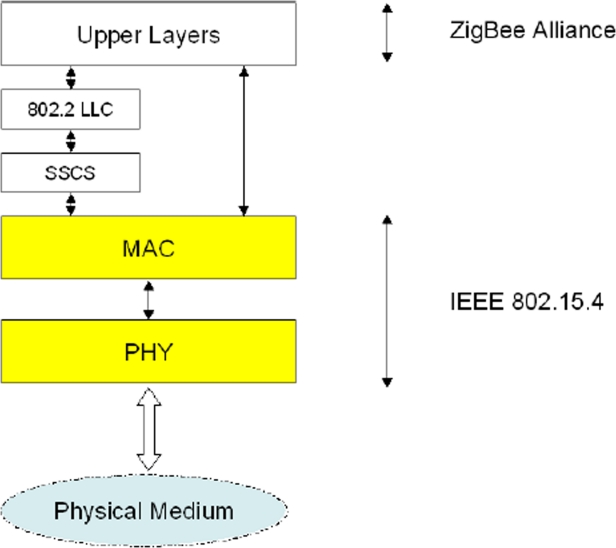
ZigBee protocol stack.

The 802.15.4 core system consists of an radio frequency (RF) transceiver and the protocol stack, depicted in Figure 5.

The 802.15.4 physical layer operates in three different unlicensed bands (and with different modalities) according to the geographical area where the system is deployed. However, spread spectrum techniques are wherever mandatory to reduce the interference level in shared unlicensed bands.

IEEE 802.15.4 specifies a total of 27 half-duplex channels across the three frequency bands and is organized as follows:

- the 868 [MHz] band: only a single channel with data rate 20 [kbps] is available; *−*92 [dBm] RF sensitivity required and ideal transmission range approximatively equal to 1 [km];
- the 915 [MHz] band: ten channels with rate 40 [kbps] are available; the receiver sensitivity and the ideal transmission range are the same of the previous case;
- the 2.4 [GHz] ISM band: sixteen channels with data rate 250 [kbps] available; minimum −85 [dBm] RF sensitivity required and ideal transmission range equal to 220 [m].

The ideal transmission range is computed considering that, although any legally acceptable power is permitted, IEEE 802.15.4-compliant devices should be capable of transmitting at −3 [dBm].

According to the energy efficiency issue, low rate and low duty cycle are provided. IEEE 802.15.4-compliant devices are active only during a short time and the standard allows some devices to operate with both the transmitter and the receiver inactive for over 99% of time.

### 5.2. IEEE 802.15.4 MAC Layer

IEEE 802.15.4 uses a protocol based on the CSMA/CA algorithm, which requires listening to the channel before transmitting to reduce the probability of collisions with other ongoing transmissions.

IEEE 802.15.4 defines two different operational modes, namely the *beacon-enabled* and the *non beacon-enabled*, which correspond to two different channel access mechanisms.

In the non beacon-enabled mode nodes use an unslotted CSMA/CA protocol to access the channel and transmit their packets [12]. The algorithm is implemented using units of time called backoff periods. First, each node will delay any activities for a random number of backoff periods. After this delay, channel sensing is performed for one unit of time: if the channel is found free the node immediately starts the transmission; if, instead, the channel is busy the node enters again in the backoff state. There exists a maximum number of time the node can try to access the channel (i.e., to sense the channel). When this maximum is reached, the algorithm ends and the transmission cannot occur.

**Figure 6.**
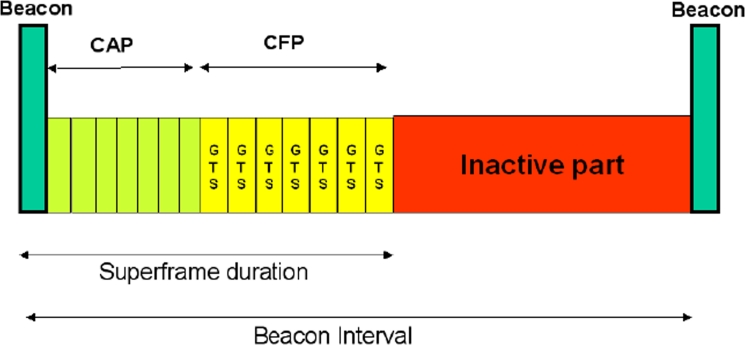
Superframe structure.

In the beacon-enabled mode [12], instead, the access to the channel is managed through a superframe, starting with a packet, called *beacon*, transmitted by WPAN coordinator. The superframe may contain an inactive part, allowing nodes to go in sleeping mode, whereas the active part is divided into two parts: the Contention Access Period (CAP) and the Contention Free Period (CFP), composed of Guaranteed Time Slots (GTSs), that can be allocated by the sink to specific nodes (see Figure 6). The use of GTSs is optional.

The duration of the active part and of the whole superframe, depend on the value of two integer parameters ranging from 0 to 14, that are, respectively, the superframe order, denoted as *SO*, and the beacon order, denoted as *BO*, with *BO* ≥ *SO*. *BO* defines the interval of time between two successive beacons, namely the beacon interval, denoted as *BI*; its duration is equal to

(1) $$\mathrm{BI}=16\cdot 60\cdot 2^{\mathrm{BO}}\cdot T_{s}$$

where *T_s_* = 16 [*μs*] is the symbol time.

The duration of the active part of the superframe, containing CAP and CFP, namely the superframe duration, denoted as *SD*, is equal to

(2) $$\mathrm{SD}=16\cdot 60\cdot 2^{\mathrm{SO}}\cdot T_{s}$$

According to the standard each GTS must have a duration multiple of 60·2*^SO^* ·*T_s_* and must contain the packet to be transmitted by the node to which the GTS is allocated to and also an inter-frame space, equal to 40 *T_s_*. This is, in fact, the minimum interval of time that must be guaranteed between the reception of two subsequent packets. The WPAN coordinator may allocate up to seven GTSs, but a sufficient portion of the CAP must remain for contention-based access. The minimum CAP duration is equal to 440 *T_s_*.

For what concerns the CSMA/CA algorithm used in the CAP portion of the superframe the only difference with the non beacon-enabled mode is that nodes have to find the channel free for two subsequent backoff periods before transmitting the packet. The other difference with the non beacon-enabled case is that backoff period boundaries of every node in the WPAN must be aligned with the superframe slot boundaries of the coordinator; therefore, the beginning of the first backoff period of each node is aligned with the beginning of the beacon transmission. Moreover, all transmissions may start on the boundary of a backoff period.

### 5.3. IEEE 802.15.4 Network Topologies and Operational Modes

To overcome the limited transmission range, multi-hop self-organizing network topologies are required. These can be realized taking into account that IEEE 802.15.4 defines two types of devices: the Full Function Device (FFD) and the Reduced Function Device (RFD). The FFD contains the complete set of MAC services and can operate as either a PAN coordinator or as a simple network device. The RFD contains a reduced set of MAC services and can operate only as a network device.

**Figure 7.**
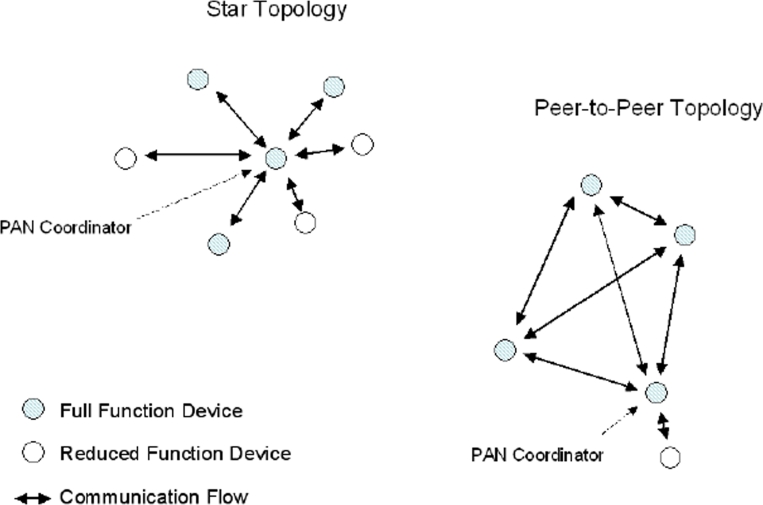
The two IEEE 802.15.4-compliant network topologies: star and peer-to-peer topology.

Two basic topologies are allowed, but not completely described by the standard since definition of higher layers functionalities are out of the scope of 802.15.4: the star topology, formed around an FFD acting as a PAN coordinator, which is the only node allowed to form links with more than one device, and the peer-to-peer topology, where each device is able to form multiple direct links to other devices so that redundant paths are available. An example of both the IEEE 802.15.4-compliant network topologies is shown in Figure 7.

Star topology is preferable in case coverage area is small and low latency is required by the application. In this topology, communication is controlled by the PAN coordinator that acts as network master, sending packets, named *beacons* for synchronization and managing device association. Network devices are allowed to communicate only with the PAN coordinator and any FFD may establish its own network by becoming a PAN coordinator according to a predefined policy. A network device that wishes to join a star network listens for a beacon message, and after receiving it, the network device can send an association request back to the PAN coordinator, which either allows or denies the association. Star networks also support a non beacon-enabled mode. In this case, beacons are used for association purpose only, whereas synchronization is achieved by polling the PAN coordinator for data on a periodic basis. Star networks operate independently from their neighboring networks.

Peer-to-peer topology is preferable in case a large area should be covered and latency is not a critical issue. This topology allows the formation of more complex networks and permits any FFD to communicate with any other FFD behind its transmission range via multi-hop. Each device in a peer-to-peer structure needs to proactively search for other network devices. Once a device is found, the two devices can exchange parameters to recognize the type of services and features each supports. However, the introduction of multihop requires additional device memory for routing tables.

IEEE 802.15.4 can also support other network topologies, such as cluster, mesh, and tree. These last network topology options are not part of the IEEE 802.15.4 standard, but are described in the ZigBee Alliance specifications [32] (see Section 5).

All devices belonging to a particular network, regardless of the type of topology, use their unique IEEE 64-bit addresses and a short 16-bit address is allocated by the PAN coordinator to uniquely identify the network.

### 5.4. The IEEE 802.15.4 Topology Formation Procedure

The IEEE 802.15.4 defined a mechanism to support a PAN coordinator in channel selection when starting a new PAN, and a procedure, called *association procedure*, which allows other devices to join the PAN. A PAN coordinator wishing to establish a new PAN needs to find a channel free from the interference that would render the channel unsuitable (e.g., in a multi-sink network, a channel may be already occupied by other PANs). The channel selection is performed by the PAN coordinator through the Energy Detection (ED) scan which returns the measure of the peak energy in each channel. It must be noticed that the standard only provides the ED mechanism, and it does not specify the channel-selection logic. The operations accomplished by a device to discover an existing PAN and to join it can be summarised as follows: (i) search for available PANs; (ii) select the PAN to join; (iii) start the association procedure with the PAN coordinator or with another FFD device, which has already joined the PAN. The discovery of available PANs is performed by scanning beacon frames broadcasted by the coordinators. Two different types of scan that can be used in the association phase are proposed:

1. passive scan: in beacon-enabled networks, the associated devices periodically transmit beacon frames, hence the information on the available PAN can be derived by eavesdropping the wireless channels;
2. active scan: in non-beacon-enabled networks, the beacon frames are not periodically transmitted but shall be explicitly requested by the device by means of beacon request command frame.

After the scan of the channels, a list of available PANs is used by the device to choose the network to try to connect with. In the standard, no specific procedure to select a PAN is provided and so, this selection among potential parents is open for different implementations. Hence, the device sends an association request frame to the coordinator device by means of which the selected network was discovered. The association phase ends with a successful association response command frame to the requesting device. This procedure basically results in a set of MAC association relationships between devices, named in the following parent-child relationship.

### 5.5. Zigbee and the Tree-Based Topology

ZigBee defines the network and application layers above the 802.15.4 [32]. Zigbee is being promoted by the Zigbee Alliance and its main contribution is giving mesh network capabilities to 802.15.4 applications. Mesh networking allows reconfiguration around blocked paths by hopping from node to node until the data reaches the destination. Moreover, Zigbee specifications define a beacon-enabled tree-based topology, as a particular case of the IEEE 802.15.4 peer-to-peer networks.

**Figure 8.**
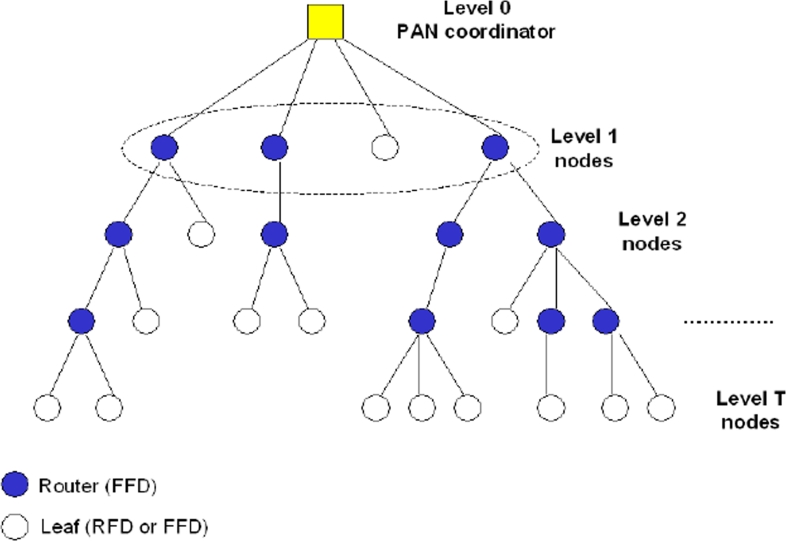
ZigBee-compliant tree network topology.

This topology, depicted in Figure 8 as an example, can be interpreted as a hierarchical tree where nodes at a given level transmit data to nodes at a lower level, to reach the PAN coordinator, which is the root of the tree. Only one device in tree assumes the role of PAN coordinator, that is generally the sink of the scenario. In case of multi-sink scenario more PAN coordinators are present and a forest of disjoint trees, rooted at the PAN coordinators is established.

Two different types of nodes are present in the tree: the routers, that must be FFDs, which receive data from their children, aggregate them, and transmit the packet obtained to their parents; and the leafs, that could be FFDs or RFDs, which have no routing functionalities and have only to transmit their packets to the parent.

The topology formation procedure is started by the PAN coordinator, which broadcasts beacon packets to neighbour nodes. A candidate node receiving the beacon may request to join the network at the PAN coordinator. If the PAN coordinator allows the node to join, it will begin transmitting periodic beacons so that other candidate nodes may join the network.

**Figure 9.**
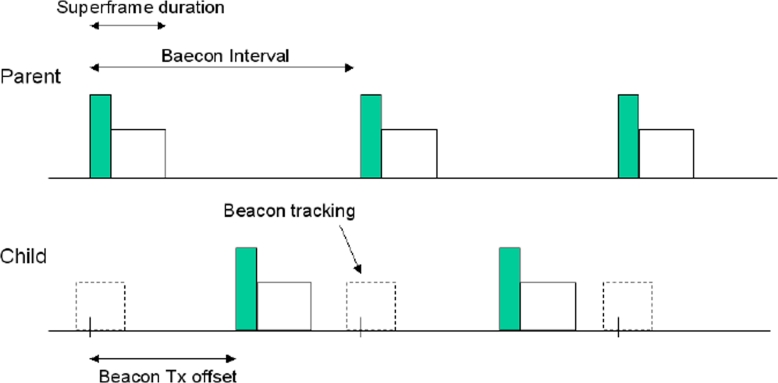
The tracking of the beacon’s parent, performed by a generic child.

As stated above, nodes must be in beacon-enabled mode: each child node tracks the beacon of its parent (see Figure 9, where the tracking period is outlined as a dashed rectangle). A core concept of this tree topology is that the child node may transmit its own beacon at a predefined offset with respect to the beginning of its parent beacon: the offset must always be larger than the parent superframe duration and smaller than beacon interval (see Figure 9). This implies that the beacon and the active part of child superframe reside in the inactive period of the parent superframe; therefore, there is no overlap at all between the active portions of the superframes of child and parent. This concept can be expanded to cover more than two nodes: the selected offset must not result in beacon collisions with neighbouring nodes. This implies that the node must record the time stamp of all neighbouring nodes and selects a free time slot for its own beacon. Obviously a child will transmit a beacon packet only when it is a router in the tree; if the child is a leaf it has only to transmit the packet to its parent. Each child will transmit its packet to the parent in the active part (CAP or CFP) of the parent superframe. Therefore, each router in the tree, after the reception of the beacon coming from the parent, will select the instant in which transmits its beacon. Beacon scheduling is necessary to prevent the beacon frames of one device from colliding with either the beacon frames or data transmissions of its neighboring devices.

**Figure 10.**
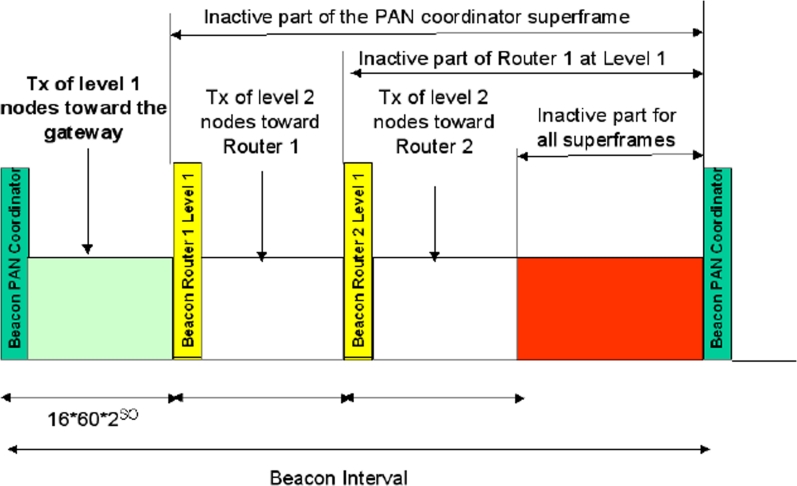
The superframe structure used in the tree-based topology.

An example of superframe structure in 3-level tree, having two routers at level one, is shown in Figure 10. Routers at level 1 transmit the beacon and define superframes that are not overlapped and all contained in the inactive part of the PAN coordinator superframe.

### 5.6. 6LowPan

In 2007 the Internet Engineering Task Force (IETF) has released an open standard called 6LowPAN in order to use IPv6 over 802.15.4. Abbreviation *6LowPAN* stands for IPv6 over Low-Power WPANs. IP for Smart Objects (IPSO) Alliance is promoting the use of 6LowPAN and embedded IP solutions in smart objects. As Zigbee bottom two layers in 6LowPAN are the 802.15.4 layers.

6LowPAN is a protocol definition describing how to utilize IPv6 on top of low power, low data rate, low cost personal area networks. The charter of 6LowPAN working group is to define how to carry IP-based communication over IEEE 802.15.4 links while conforming to open standards and assuring interoperability with other IP devices. Some key technologies of 6LowPAN are as follows [33]. (i) The coordination of IPv6 and IEEE802.15.4: by defining an adaptation layer, 6LowPAN compresses the 60 bytes long headers in IPv6 to 7 bytes and fragments the 1280 bytes long IPv6 packets to fit 127 bytes long 802.15.4 packets. (ii) Address assignment and management: one of the distinctive features of 6LowPAN is the capability of the dynamic assignment of 16-bit short addresses; by using this short address, hierarchical routing can be employed. (iii) Network management: owing to the large scale of network and the distribution of place, LR-WPAN should possess self-healing ability, and LR-WPAN management technology is requested to be able to manage highly dense deployment equipments with a very low expense. 6LowPAN is apt to use SNMPv3 (Simple Network Management Protocol) in LR-WPAN to progress network management.

The fundamental difference between 6LowPAN and Zigbee is the IP interoperability of the first. 6LowPAN devices are capable of communication with other IP-enabled devices whereas Zigbee node needs an 802.15.4/IP gateway to interact with an IP network. The decision to select one standard versus another should be determined by the target application. For an application in which there is no need to interface with IP devices or the packet size is small, it is not necessary to implement 6LowPAN, which performs fragmentation. Zigbee can achieve better overall performance in such an application.

### 5.7. Performance Trends of 802.15.4 Based WSNs

In this section some examples of performance trends obtained from the study of IEEE 802.15.4 networks are shown. The aim is to provide some numerical results in terms of throughput and energy consumption and to show how the choice of the topology affects performance in WSNs. Results are achieved through experimental measurements, using Freescale devices IEEE 802.15.4-compliant working at 2.4 GHz [34], and through the mathematical models of non-beacon- and beacon-enabled 802.15.4 networks described in [35] and [36], respectively.

**Figure 11.**
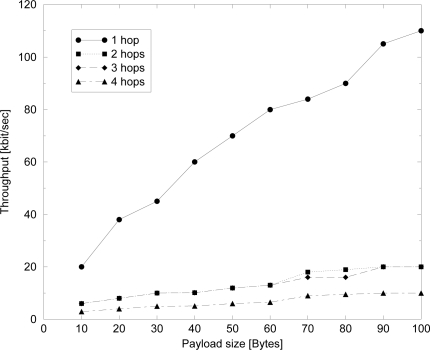
Throughput measured for a point-to-point 802.15.4 network when one, two three or four routers are present.

In Figure 11 experimental results are shown. A point-to-point network, where a source node has to transmit data to a destination node, possibly through a number of routers, is considered. When one router between the source and the destination is present, a two-hop communication is performed; in case of two routers we have three hops, etc. Nodes work in beacon-enabled mode, and we set *SO* = *BO* = 0 for the one-hop case and *SO* = 0 and *BO* = 2 for the multi-hop cases. The figure shows the behavior of the throughput, that is the number of bits (of the MAC payload) per second successfully received by the final destination, as a function of the payload size. Even if the channel bit rate is 250 kbit/sec, the throughput is significantly smaller because of the protocol overhead, mainly due to the MAC layer. As we can see the throughput does not reach more than about 120 kbit/sec for point-to-point links. However, the throughput can be significantly lowered owing to the potentially interference among the separate hops disturbing each other.

**Figure 12.**
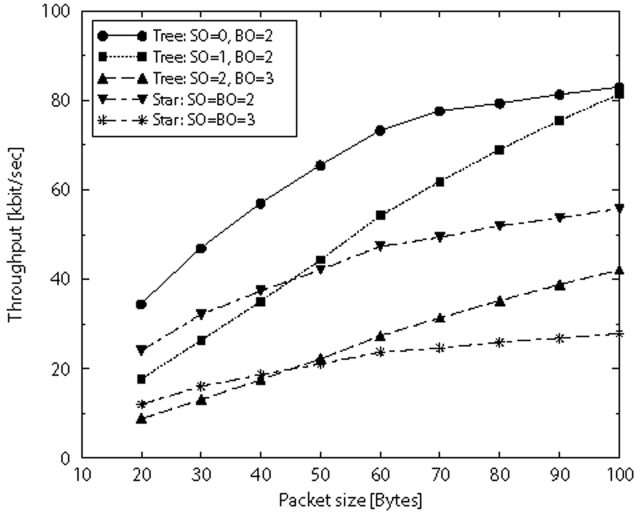
Throughput as a function of packet size for an 802.15.4 network organised in star and tree-based topologies.

In Figure 12 a single-sink scenario where 802.15.4 nodes transmit data to the sink through one link (star topology), or possibly two-hops (3-level tree, rooted at the sink), is considered. A network composed of 30 nodes, working in beacon-enabled mode is accounted for. In the figure we show the throughput as a function of the size of the packets transmitted by nodes. The throughput here represents the number of bits (of the payload) per second correctly received by the sink when all the 30 nodes try to access the channel and transmit their packets, assuming that nodes transmit packets of the same size.

For a fair comparison in terms of delay, curves obtained by setting the same value of *BO* should be considered (meaning that the superframes have the same whole duration). As we can see, for low values of the packet size, stars outperform trees, whereas trees perform better when the packet size increases, since less nodes compete to access the channel at the same time (nodes are split in two levels). The best case is obtained in case of “tree” when *SO* = 0 and *BO* = 2. This is, in fact, for the network chosen (with 30 nodes and a mean number of 3 level one nodes) the best compromise between the duration of the active part of the sink superframe (where level one nodes transmit) and that of the inactive part (where level 1 superframes are located).

The differences between results in Figure 11 for the case two-hop and those in Figure 12 for the tree case when *SO* = 0 and *BO* = 2 are due to the fact that in the first case only one node is transmitting toward the sink, whereas in the latter case 30 nodes are competing for the channel. The increase of the number of nodes on one hand increases the amount of data transmitted toward the sink per unit of time, but, on the other, the success probability, that is the probability that a node succeeds in transmitting correctly the packet, decreases [36].

**Figure 13.**
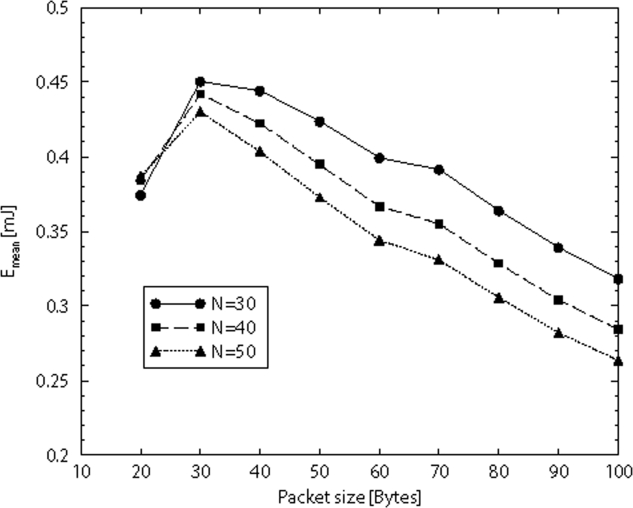
The mean energy spent by an 802.15.4 node working in non beacon-enabled mode, belonging to a network of *N* nodes organised in a star topology.

Finally, in Figure 13 the mean energy spent by a single node having a packet of a given size to be transmitted, is shown. We assume that the network is composed of *N* 802.15.4 nodes working in non beacon-enabled mode and competing to transmit their packets to the sink through a direct link. The model developed in [35] is used. The behavior of the mean energy spent as a function of the packet size, for different values of *N*, is shown. Here we refer to the whole packet size, composed of the PHY and MAC headers (of 15 bytes) and the payload. As we can see, the mean energy spent presents a maximum. This is due to the fact that for low packet sizes, the increase of the size increases the energy as well, since a larger amount of energy is spent for transmitting larger packets. Conversely, when the packet size becomes too large, the energy starts to decrease since the probability that the node succeeds in accessing the channel and transmitting its packet gets lower as well.

## 6. Other Technologies

### 6.1. Ultrawide Bandwidth Technology

Ultrawide bandwidth radio is a fast emerging technology with uniquely attractive features that has attracted a great deal of interest from academia, industry, and global standardization. bodies. The most widely accepted definition of a UWB signal is a signal with instantaneous spectral occupancy in excess of 500 MHz or a fractional bandwidth of more than 20%. One of the most promising UWB techniques, especially for WSN applications, is named Impulse Radio-UWB (IR-UWB) [37, 38]. The IR-UWB technique relies on ultra-short (nanosecond scale) waveforms that can be free of sine-wave carriers and do not require IF processing because they can operate at baseband. The IR-UWB technique has been selected as the PHY layer of the IEEE 802.15.4a Task Group for WPAN Low Rate Alternative PHY layer [39]. The baseline of 802.15.4a is based on two optional PHYs consisting of a UWB impulse radio (operating in unlicensed UWB spectrum) and an other option operating in unlicensed 2.4 GHz spectrum, where the former will be able to deliver communications and high precision ranging.

### 6.2. Bluetooth Technology

Bluetooth wireless technology is a short-range communication system intended to replace the cables in WPANs (see also the web site: http://www.bluetooth.com). The key features of Bluetooth wireless technology are robustness, low power, and low cost. Many features of the core specification are optional, which allows product differentiation.

The IEEE Project 802.15.1 [40] has derived a WPAN standard based on the Bluetooth v1.1 Foundation Specifications (see also the website: http://www.ieee802.org/15/pub/TG1.html).

The Bluetooth RF (physical layer) operates in the unlicensed ISM band, for the majority of countries around 2.4 GHz in (2400, 2483.5) MHz. 79 frequency channels spacing of 1 MHz in the ISM band (e.g., *f* = 2402 + *k* MHz, with *k* = 0, . . ., 78), are available. The system employs a frequency hop transceiver (the nominal hop rate is 1600 hops/s) to combat interference and fading. RF operation uses a Gaussian shaped, binary frequency shift keying (GFSK) modulation to minimize transceiver complexity, and a forward error correction (FEC) coding technique. The bit rate is of 1 Mbps.

Nodes are organized in piconets, managed by a master node and having a maximum number of seven active slaves. The physical channel is sub-divided into time units known as slots with duration 625 *μ*s. Bluetooth technology provides the effect of full duplex transmission through the use of a time-division duplex (TDD) scheme.

### 6.3. Z-Wave

Z-Wave is a technology developed by the Danish company Zensys; it uses a low-power RF radio for low-power remote control applications. The technology has been standardized by the Z-Wave Alliance. This technology is not compatible with 802.15.4. The main advantage of this technology with respect to 802.15.4 is its operation in sub 1 GHz band. The 2.4 GHz RF band, is fact, is subject to significant interference due to 802.11 and 802.15.1 devices. On the other hand the 868 MHz ISM band used by Z-Wave is limited by European regulations to operate at or under 1%. However 1% duty cycle operation in the band can be enough for most of the control applications. Mesh topologies could be formed, however the addressing scheme used allows a maximum of 232 nodes in the network. Operable data rates are 9.6 kbps and 40 kbps. The transceivers from Zensys allow 100 meters of outdoor range.

### 6.4. Bluetooth Low Energy Technology

The developing process of this ultra-low-power Bluetooth technology is started within the FP6 funded project MIMOSA. The technology was released to public in October 2006 with the name “Wibree”. Basically it is the simplified version of Bluetooth. It uses the same physical layer in 2.4 GHz ISM used by Bluetooth for interoperation with existing Bluetooth devices and allows 1 Mbit/s data rates in up to 10 meters range. Bluetooth low energy is designed to be very efficient at transmitting very small quantities of data at very low latencies to other devices. When compared to classic Bluetooth technology it is maximum 15 times more efficient. It achieves these efficiency gains by optimizing three basic areas of functionality: connectable and discoverable modes, the number of packets transmitted during connections, and the size of each individual packet.

In classic Bluetooth technology, for a device to be connectable or discoverable it must enable its receiver. Therefore, the only way to be responsive is to have the radio active for a significant period of time. A basic requirement for two frequency hopping devices to communicate is that they need to use the same frequency or channel at the same time, i.e., they need to be synchronized. When the devices first start communicating, they are not synchronized, and they need to search different channels to find each other. In Bluetooth technology, 32 channels are used. Searching through that many channels takes time, and in Bluetooth technology it can take up to a couple of seconds for two devices to find each other, which consumes power. In Bluetooth low energy technology, instead, there are only three channels used for advertising. This brings Bluetooth low energy to be over 17 times more efficient than classic Bluetooth.

There are two other major differences between the two version of the standard: Bluetooth low energy uses fewer channels, and the hop sequence used by the radios is different. There are two reasons why there are fewer channels: Bluetooth low energy uses a larger modulation index, meaning that its signal takes up more bandwidth, and has relaxed requirements for how steep the channel filters need to be. Because of this, Bluetooth low energy channels are spaced 2 MHz apart, rather than 1 MHz apart as in Bluetooth technology. Both of these design choices were made in order to provide a lower power consumption.

Another important improvement of the low energy version, is that when a slave device does not have any data to transmit, it does not even have to bother listening to the master device’s communication event packets. This enables the slave device to stay in the lowest possible power mode for as long as possible, further saving significant amounts of power. However, if it does have something important to transmit, then it can wake up at the next appropriate communication event and transmit its data very quickly. This enables an excellent compromise between ultra low power operation and low latency transmission of data.

Regarding the network topology, instead, unlike Zigbee, 6LowPAN, and Z-Wave, it does not support mesh networking.

Development of the technology is currently under way. Texas Instruments and Nordic Semiconductor are developing compatible devices but transceivers are not on market yet.

### 6.5. ANT Technology

ANT is a proprietary technology featuring a wireless communication protocol stack thought for ultra-low power networking applications. It is designed to run using low cost, low power microcontrollers and transceivers operating in the 2.4 GHz ISM band. The ANT WSN protocol has been engineered for simplicity and efficiency, resulting in an ultra-low power consumption, maximised battery life, a minimal burden on system resources, simpler network designs and lower implementation costs. ANT also features low latency, the ability to trade-off data rate against power consumption, and support for broadcast, burst and acknowledged transactions up to a net data rate of 20 kbit/s (ANT’s over the air data rate is 1 Mbit/s). Different topologies could be established: peer-to-peer, star, tree and other types of mesh network.

ANT nodes are capable of acting as slaves or masters within a network and swapping roles at any time. This means the nodes can act as transmitters, receivers or transceivers to route traffic to other nodes. ANT is a good protocol for practical networks because of this inherent ability to support ad hoc interconnection of tens or hundreds of nodes.

ANT allows a system to spend most of its time in an ultra-low power sleep mode, wake up quickly, transmit for the shortest possible time and quickly return back to an ultra-low power sleep mode. This implies that ANT is one of the most energy-efficient available technologies. While Bluetooth is designed for rapid file transfer between devices in a PAN, its average power consumption is 10 times greater with respect to ANT and the hardware costs are 90 percent higher. With respect to IEEE 802.15.4 ANT presents a larger data rate of 1 Mbit/sec and is relatively less complex.

However, being a proprietary technology, ANT lacks interoperability.

## 7. Future Research Directions and Projects

Basically, the research in the field of WSNs started very recently with respect to other areas of the wireless communication society, as examples like broadcasting or cellular networks. The first IEEE papers on WSNs were published after the turn of the Millennium.

The first European projects on WSNs were financed after year 2001: During the sixth and seventh Framework Programmes, some Projects were financed by the EC, with explicit activities dedicated to communication protocols, architectural and technological solutions for embedded systems: among them, the first to be launched were WISENTS [41], e-SENSE [42], CRUISE [43] and CONET [44]. In the US the research on WSNs was boosted few years before.

Standardization is a key issue for success of WSN markets. The possible options for building HW/SW platforms for WSNs have been identified in the previous section. For low data rate applications (250 kbit/s on the air), IEEE 802.15.4 seems to be the most flexible technology currently available, while also Bluetooth LE can be attractive for applications demanding higher data rates. However, IEEE is also currently developing a new standard specifically oriented to WSNs for Body Area Networks, through the Task Group 802.11.6. While this shows the perceived relevance of standards in the research arena, it will also set the basis for the possible creation of an heterogeneous WSN environment, and opens the field to new technical solutions: in fact, many technical topics of WSNs are still considered by research, as the current solutions are known to be non optimized, or too much constrained.

Then, it is also relevant to mention that there exist two European Technology Platforms, gathering all stakeholders in the field, related to the area of WSNs: e-Mobility and ARTEMIS. They have drawn research agendas that will drive the selection of large cooperative projects in the next years in Europe.

In particular, ARTEMIS (Sub-Programme 7) is currently financing (2009–2011) the project eDIANA (Embedded Systems for Energy Efficient Buildings), addressing the need of achieving energy efficiency in buildings through innovative solutions based on embedded systems, described in Section 3.

For a proper discussion on future WSN research directions, it is useful to split the issue in two: some research efforts are in fact application-agnostic, while others are mainly driven by market trends and future application needs.

### 7.1. Application-Agnostic Research Trends

From the physical layer viewpoint, clearly the need to have low-complexity and low-cost devices does not push short term research in the direction of advanced transmission techniques, while in the medium-long term the application of some novel concepts such as *cognitive radio networking* might be applied to WSNs. The wide use of unlicensed bands for WSNs bring to the situation that they often need to be deployed in environments where many other wireless devices operate, e.g., WiFi systems. A proper use of the radio resource in such unplanned and dynamic environment requires the ability to adapt transmission techniques to the current use of the spectrum. Therefore, some adaptability to the spectrum usage needs to be implemented in future platforms for WSNs.

MAC and network layer have attracted a lot of attention in the past years and still deserve investigation. In particular, combined approaches that jointly consider MAC and routing seem to be very successful. Once more, making MAC and routing protocols spectrum-aware can bring to relevant performance advancements in some environments.

Topology creation, control and maintenance are very hot topics. Especially with IEEE 802.15.4, which allows creation of several types of topologies (stars, mesh, trees, cluster-trees), these issues play a very significant role.

One more general paradigm that will be applied to the field of WSN is *Cooperation*, defined as the ability of individual entities or objects (that could be sensors, controllers or actuators) to use communication as well as dynamic and loose federation to jointly strive to reach common goal while taking care not to overtax their available resources [44].

### 7.2. Market- and Application-Driven Research Trends

The development of WSN solutions require significant effort in terms of tailoring of the available HW/SW platforms to the specific needs of applications. Therefore, the development/deployment costs can be very high, if the market size is small. As a consequence, the most successful applications of WSN technology will be those oriented to applications including large number of nodes.

Large number of nodes require the presence of many contexts of similar nature where the same technology/application can be deployed. As examples, for this discussion, we consider buildings, humans, and vehicles; all these “contexts” exist in the current world in large numbers and as such they represent huge potential markets.

- Buildings; monitoring and control of building energy efficiency is, as also described formerly, one of the most relevant applications for WSNs in the short term. The sensing of energy consumption in residential buildings require the installation of sensor nodes in each electric appliance, counting up to tens of devices per residential unit. In a large building, hundreds or even thousand of nodes might be deployed, and interference and network management issues might be based on complex approaches. Scale factors might be introduced in the market because of these large numbers, bringing to significant cost decreases. In this scenario, part of the nodes might be networked through wired connections using the electric grid, and part by means of the technology of WSNs. The application of cognitive radio networking concepts, mentioned above, might be very useful in such dynamic and unpredictable interference contexts.
- Humans; Body Area Networks for health applications are one of the emerging markets for WSNs. However, other applications might be considered dealing with humans: inter-body communication networks might be of interest in several fields of applications, ranging from mood-based services for human networking, or in specific professional contexts. This type of application requires the exchange of personal body-generated information among humans that can interact for few seconds while walking or moving. Therefore, many challenges raise from the viewpoint of the ability of the WSN to fast react to new requests of association, etc.
- Vehicles; in this context, two types of applications might be considered. First, those were vehicles download data from fixed sensors which need to be used on-board for transportation purposes. Second, those were fixed sensors upload the sensed data on vehicles passing by, with the opportunistic goal of having such information carried to the final destination exploiting the partially predictable movements of vehicles. The latter concept is sometimes mentioned as *opportunistic networking* and is one of the emerging paradigms of future wireless networks; only delay tolerant applications can be run, with the advantage of the efficient exploitation of all types of resources available in the environment where the WSN is deployed.

## 8. Conclusions

The aim of this paper is to discuss some of the most relevant issues of WSNs, from the application, design and technology viewpoints. For designing a WSN, in fact, we need to define the most suitable technology to be used and the communication protocols to be implemented (topology, signal processing strategies, etc.). These choices depend on different factors, above all the application requirements. The first part of the paper is devoted to the discussion on the constraints that must be satisfied by the WSN and the different aspects that must be taken into consideration in the design of a WSN. The second part, instead, is related to the actual possible choices that could be done, in terms of technologies. The aim is to help the designer in the choice of the most suitable technology. The attention is mainly focused on the IEEE 802.15.4 standard, for which also some potential performance levels are provide. Finally, the paper provides a vision on future trends of the short- and long-term research on WSNs.
